# Defining indocyanine green fluorescence to assess anastomotic perfusion during gastrointestinal surgery: systematic review

**DOI:** 10.1093/bjsopen/zraa074

**Published:** 2021-04-24

**Authors:** M D Slooter, M S E Mansvelders, P R Bloemen, S S Gisbertz, W A Bemelman, P J Tanis, R Hompes, M I van Berge Henegouwen, D M de Bruin

**Affiliations:** 1 Departments of Surgery, Amsterdam the Netherlands; 2 Biomedical Engineering and Physics, Amsterdam University Medical Centre, University of Amsterdam, Amsterdam, the Netherlands

## Abstract

**Background:**

The aim of this systematic review was to identify all methods to quantify intraoperative fluorescence angiography (FA) of the gastrointestinal anastomosis, and to find potential thresholds to predict patient outcomes, including anastomotic leakage and necrosis.

**Methods:**

This systematic review adhered to the PRISMA guidelines. A PubMed and Embase literature search was performed. Articles were included when FA with indocyanine green was performed to assess gastrointestinal perfusion in human or animals, and the fluorescence signal was analysed using quantitative parameters. A parameter was defined as quantitative when a diagnostic numeral threshold for patient outcomes could potentially be produced.

**Results:**

Some 1317 articles were identified, of which 23 were included. Fourteen studies were done in patients and nine in animals. Eight studies applied FA during upper and 15 during lower gastrointestinal surgery. The quantitative parameters were divided into four categories: time to fluorescence (20 studies); contrast‐to‐background ratio (3); pixel intensity (2); and numeric classification score (2). The first category was subdivided into manually assessed time (7 studies) and software‐derived fluorescence–time curves (13). Cut‐off values were derived for manually assessed time (speed in gastric conduit wall) and derivatives of the fluorescence–time curves (F_max_, T_1/2_, TR and slope) to predict patient outcomes.

**Conclusion:**

Time to fluorescence seems the most promising category for quantitation of FA. Future research might focus on fluorescence–time curves, as many different parameters can be derived and the fluorescence intensity can be bypassed. However, consensus on study set‐up, calibration of fluorescence imaging systems, and validation of software programs is mandatory to allow future data comparison.

## Teaser

**Antecedentes:**

El objetivo de esta revisión sistemática fue identificar todos los métodos para cuantificar una angiografía de fluorescencia (*fluorescence angiography*, FA) intraoperatoria en anastomosis gastrointestinales y encontrar umbrales que pueden predecir los resultados en el paciente, incluida la fuga anastomótica y la necrosis.

**Métodos:**

Esta revisión sistemática se adhirió a las normas PRISMA (*Preferred Reporting Items for Systematic Reviews and Meta‐Analyses guidelines*). Se realizó una búsqueda bibliográfica en PubMed y Embase. Se incluyeron aquellos artículos en los que se realizó una FA con verde de indocianina para evaluar la perfusión gastrointestinal, en humanos o animales, y se analizó la señal de fluorescencia utilizando variables cuantitativas. Se definió una variable como "cuantitativa" cuando potencialmente se podía establecer un umbral numérico diagnostico respecto a los resultados del paciente.

**Resultados:**

Se identificaron 1.317 artículos, de los que se incluyeron 23. Catorce estudios se habían efectuado en seres humanos y 9 en animales. Ocho estudios realizaron la FA durante cirugías gastrointestinales altas y 15 durante cirugías gastrointestinales bajas. Las variables cuantitativas se dividieron en cuatro categorías: (1) tiempo hasta la fluorescencia (*n* = 20), (2) relación con el contraste de fondo (*n* = 3), (3) intensidad de píxeles (*n* = 3) y (4) puntuación numérica clasificatoria (*n* = 2). La primera categoría se subdividió en (1a) cuando el tiempo hasta la fluorescencia se evaluó manualmente (*n* = 7) y (1b) cuando se realizaron curvas de tiempo de fluorescencia mediante programas informáticos (*n* = 13). Los valores de corte para predecir los resultados en el paciente se obtuvieron para el tiempo evaluado manualmente (velocidad en la pared del conducto gástrico) y para el estimado mediante curvas de fluorescencia (Fmáx, T1 / 2, TR y pendiente).

**Conclusión:**

El tiempo hasta la fluorescencia parece ser la variable más prometedora para la cuantificación de la FA. La investigación futura debería centrarse en las curvas de tiempo hasta la fluorescencia, ya que de ellas se pueden derivar muchas variables diferentes y se puede evitar la intensidad de fluorescencia. Sin embargo, es obligado llegar a un consenso sobre la configuración del análisis, la calibración de los sistemas de imágenes de fluorescencia y la validación de los programas informáticos para permitir, en el futuro, la comparación de datos.

The purpose of this systematic review was to provide an overview of all methods used to quantify fluorescence angiography of the gastrointestinal anastomosis, and to identify a threshold that could predict patient outcomes, including anastomotic leakage and necrosis. Four categories of quantitative parameter were identified: time to fluorescence; contrast‐to‐background ratio; pixel intensity; and numeric classification score. Cut‐off values were derived only for the first category; thus, time to fluorescence seems the most promising method for quantitation of fluorescence angiography.

Time for standardisation

## Introduction

Anastomotic leakage (AL) remains one of the most severe complications after gastrointestinal cancer surgery with restoration of continuity. Leakage rates of up to 20 per cent are reported after restorative cancer resection of both the upper and lower gastrointestinal tract^1^, ^2^. Various risk factors have been associated with AL^3^, ^4^. Adequate blood perfusion has been described as one of the key factors for adequate healing of the anastomosis, and is a surgically modifiable factor.

To aid the surgeon with assessment of gastrointestinal perfusion and determination of the optimal site for anastomosis, fluorescence angiography (FA) has gained support among gastrointestinal surgeons^5^, ^6^. FA is a technique that uses an imaging system capable of excitation and detection of the fluorescent contrast agent indocyanine green (ICG)^7^. ICG is a cyanine dye with an absorption and emission peak in the near‐infrared region^6^, at about 800 nm. ICG is approved for FA by the US Food and Drug Administration and European Medicines Agency, and is safe to use as side‐effects occur rarely^6^. After intravenous injection, ICG distributes through the vascular system bound to plasma proteins, and its immediate fluorescence detection correlates with areas of perfused tissue. ICG is detectable within 1 min of injection^5^, and imaging can be performed in real time, making FA suitable for intraoperative enhanced reality of perfusion. This aids the surgeon in optimizing the anastomotic site and potentially lowering AL secondary to insufficient perfusion. In early observations^8^, ^9^, ^10^, use of FA was reported to lower AL rates after gastrointestinal cancer surgery.

When intraoperative management was adapted according to subjective interpretation of FA, AL and perianastomotic necrosis still occurred^8^, ^9^, ^10^. Although the pathophysiology of AL is multifactorial and dependent on several factors other than perfusion, other explanations for these observations include undertreatment and overtreatment, and difficulty in visualizing venous congestion by FA. Hitherto, no threshold is known for adequate perfusion. Overtreatment might be a result of more extended resections based on the FA findings when the imaging system was not sufficiently specific for detection of ischaemia. Overtreatment can come at the cost of a tension‐free anastomosis, risking AL. Furthermore, venous congestion is more difficult to detect by subjective interpretation of FA, as ICG enters the tissue of interest when arterial blood flow is intact^11^.

To overcome the limitations of subjective interpretation of FA and evaluate ICG fluorescence objectively, research in the past decade has focused on measuring the fluorescent signal in quantitative values. However, no consensus exists on the method of quantification of the ICG fluorescence, and no threshold for adequate perfusion has yet been identified. This systematic review of the literature aimed to provide an overview of all the methods of FA quantification employed during gastrointestinal surgery and thresholds that have been produced to predict patient outcomes, in particular AL and necrosis. According to the identified methods, the aim was to outline recommendations for future research strategies.

## Methods

The authors adhered to the PRISMA guideline^12^. PubMed and Embase databases were searched on 15 January 2019 to identify all studies that performed FA during gastrointestinal surgery and investigated quantitative fluorescence values (*Appendix* *S1*, supporting information). After removal of duplicates, title and abstract screening was executed independently by two authors according to predetermined criteria (*Table* *S1*, supporting information). Subsequently, full‐text screening was conducted, and articles were deemed eligible when they presented original work on FA during gastrointestinal surgery in humans or animals. Reference lists of included articles were scanned to obtain potential additional articles. Conflicts were discussed to reach consensus.

Reported outcomes had to include a quantitative fluorescence parameter, ideally correlated with patient outcomes. A parameter was considered quantitative when a diagnostic numeric threshold for AL or necrosis could potentially be produced. Examples of quantitative fluorescence parameters are numeric classification scores, time to fluorescence enhancement, equations, or software analyses. Descriptive grouping using ‘no’, ‘little’ or ‘good’ fluorescence was not considered as numeric quantification of fluorescence.

Quality assessment of all included articles was performed independently by two authors. For human studies, the Newcastle–Ottawa Scale (NOS) for cohort studies was used. Animal studies were assessed using the SYRCLE (SYstematic Review Center for Laboratory animal Experimentation) risk‐of‐bias tool^13^.

Data extraction and aggregation was done by two authors. From all articles, only the groups that received FA were extracted and analysed for the purpose of the present review. Extracted data on the method of FA included the dose of ICG, the near‐infrared imaging system and the software program used. The primary outcome was the quantitative fluorescence parameter (one or multiple). Secondary outcomes included patient outcomes, such as AL and necrosis rates, and change in management due to FA following conventional assessment of perfusion.

To aggregate the software‐derived fluorescence–time curves, the curves were extracted from the individual graphs into data points using CurveSnap version 1 (Xoofee; https://curvesnap.en.softonic.com/) To compare the curves, the data points from the curves were read into Excel® (Microsoft, Redmond, Washington, USA). Fluorescence intensity values were normalized from 0 to 100 per cent, with the background intensity of the graph set as the lowest level of the amplitude (0 per cent) and the highest fluorescence intensity as the maximum amplitude (100 per cent). Subsequently, the curves were shifted manually to match the *t* = 0 position, which was determined as the start of increase of the ICG fluorescence curve from its background.

### Statistical analysis

Results are presented using descriptive statistics. Categorical data are presented as number of cases and percentages. Continuous data, when normally distributed, are shown as mean(s.d.) values or total range, or, when not normally distributed, as median (i.q.r.) values or total range.

## Results

**Fig. 1:**
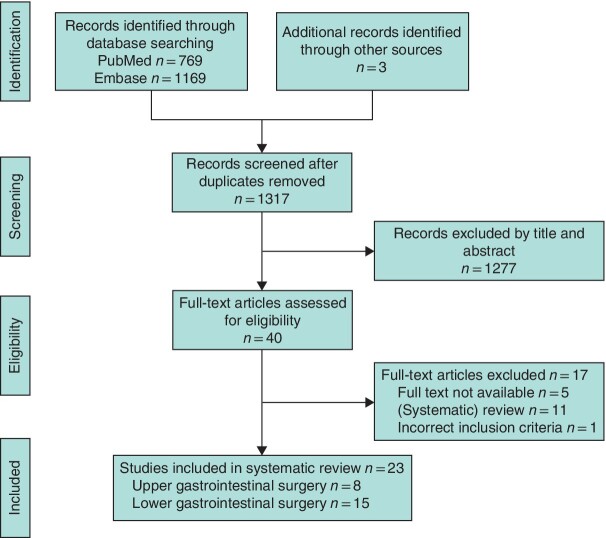
PRISMA diagram for the review

A total of 1317 records were screened for title and abstract, after which the full texts of 40 articles were assessed for eligibility. In total, 23 articles^14–36^ were included in this review, of which eight concerned upper and 15 lower gastrointestinal surgery. The process of screening and eligibility assessment is summarized in a PRISMA diagram (*Fig*. 1).

Fourteen studies were performed in humans and nine in animals. The quality of the human studies was either poor or good (*Table* *S2*, supporting information). Most studies had a non‐comparative design, so no points were granted to the comparability domain, which resulted in poor quality according to the NOS. All animal studies addressed attribution and reporting bias according to the SYRCLE classification, but selection, performance and detection bias were scarcely considered (*Table* *S3*, supporting information). Characteristics of all included studies on patients are shown in *Table* *S4* (supporting information) and those for animal studies in *Table* *S5* (supporting information); overall, 11 different imaging systems and 11 different software programs were described. Clinical outcomes were reported in 13 of the 14 articles in patients (*Table* 1).

**Table 1:** Clinical outcomes in human studies

Reference	Basis of change in management	Change in management	Anastomotic leakage	Necrosis
**Upper GI tract**				
Huh *et al*.^17^	Conventional assessment	0 of 30 (0)	1 of 30 (3)	n.a.
Ishige *et al*.^21^	FA	6 of 20 (30)	0 of 20 (0)	n.a.
Kamiya *et al*.^23^	Doppler ultrasonography and FA	5 of 26 (19)	n.a.	2 of 26 (8)
Koyanagi *et al*.^14^	FA	0 of 40 (0)	7 of 40 (18)	n.a.
Kumagai *et al*.^15^	Time from first fluorescence root RGEA to anastomotic site > 60 s	18 of 70 (26)	1 of 70 (1)	n.a.
Yukaya *et al*.^22^	n.r.	n.a.	9 of 27 (33)	n.a.
**Lower GI tract**				
Foppa *et al*.^35^	FA	4 of 160 (2·5)	n.a.	1 of 4 (25)
Kim *et al*.^18^	FA	30 of 310 (9·7)	2 of 310 (0·6)	n.a.
Kudszus *et al*.^25^	FA	28 of 201 (13·9)	7 of 201 (3·5)	n.a.
Protyniak *et al*.^36^	Conventional assessment	4 of 77 (5)	2 of 77 (3)	n.a.
Sherwinter *et al*.^19^	Conventional assessment	2 of 20 (10)	2 of 20 (10)	n.a.
Son *et al*.^26^	n.r.	n.a.	5 of 86 (6)	1 of 86 (1)
Wada *et al*.^20^	FA	18 of 112 (16·1)	5 of 112 (4·5)	n.a.

Values in parentheses are percentages. GI, gastrointestinal; n.a., not applicable; FA, fluorescence angiography; RGEA, right gastroepiploic artery; n.r., not reported.

All 23 studies reported one or more quantitative fluorescence parameters of FA (*Table* 2). For the scope of this review, the reported quantitative fluorescence parameters were divided into four categories: time to fluorescence (20 studies), including manually assessed time to fluorescence (7) and software‐derived fluorescence–time curves (13); contrast‐to‐background ratio (CBR) (3); pixel intensity (2); and numeric classification score (2).

**Table 2:** Quantitative fluorescence parameters

Reference	Parameters	Units	Overall mean(s.d.) value of parameters	Cut‐off value*
**Time to fluorescence**				
Manually assessed time				
Huh *et al*.^17^	Time to first visible fluorescence signal	min	4·1(3·2)	n.a.
Kim *et al*.^18^	Time to first visible fluorescence signal	s	37(16)	n.a.
Koyanagi *et al*.^14^	Flow speed of ICG fluorescence	cm/s	2·8(1·3)	1·76 cm/s (OR 36·5)
Kumagai *et al*.^15^	Time from first fluorescence root from RGEA to anastomotic site	s	35·3 (total range 13·0–204·0)	Anastomotic site in area perfused < 90 s (preferably < 60 s)
Quan *et al*.^16^	Time to first visible fluorescence signal	s	138·0(82·1)	n.a.
Sherwinter *et al*.^19^	Time to maximum fluorescent excitation	s	33·0(1·82)	n.a.
Wada *et al*.^20^	Time to first visible fluorescence signal	s	39 (total range 20–120)	n.a.
Software‐derived fluorescence–time curves†				
Bornstein *et al*.^24^	Rate of change of maximum (timing map), maximum pixel intensity (perfusion map)	n.r.	n.r.	n.a.
Diana *et al*.^28^–^31^	F_max_, T_max_, slope (perfusion cartogram)	AU, s, AU/s	T_max_ 5·69(3·68)	n.a.
Ishige *et al*.^21^	F_max_, T_max_	AU, s	84·9(28·2), 18·9(6·5)	n.a.
Kamiya *et al*.^23^	F_max_, T_1/2_	AU, s	n.r.	T_1/2_ 9·2 s (0·82; 80; 92)
Kudszus *et al*.^25^	Curve	AU, s	n.r.	n.a.
Nerup *et al*.^32^	F_bg_, F_max_, T_max_, slope, F_norm_	AU, s, AU/s	n.r.	n.a.
Nerup *et al*.^27^	F_norm_	AU, s	n.r.	n.a.
Son *et al*.^26^	F_bg_, F_max_, F_norm_, T_1/2_, T_max_, TR, slope	AU, s, AU/s	10·6(1·0), 58·0(3·4), n.r., 11·7(0·8), 30·3(2·3), 0·4(0·0), 2·5(0·2)‡	T_1/2_ 18 s (0·963; 100; 83·7)
				TR 0·6 (0·929; 83·3; 96·3)
				Slope 0·7 AU/s (0·123; 66·7; 92·5)
Wada *et al*.^20^	F_max_, T_1/2_, T_max_, slope	AU, s, AU/s	91·4(31·9), 12·5(7·6), 32·8(15·9), 3·6(2·2)‡	F_max_ 52·0 AU (n.r.; 100; 92·5) Slope 2·1 AU/s (n.r.; 100; 75·7)
Yukaya *et al*.^22^	F_bg_, F_max_, F_norm_, T_bg_, T_max_, T_out_ 80%	AU, s	T_bg_ 41·7(2·4)	n.a.
**Contrast‐to‐background ratio**				
Ashitate *et al*.^33^	CBR over time (F_norm_/F_bg_)	n.a.	n.r.	n.a.
Matsui *et al*.^34^	CBR over time (F_norm_/F_bg_)	n.a.	n.r.	n.a.
Quan *et al*.^16^	Ratio of gastric conduit CBR/oesophageal CBR	n.a.	0·97(0·024)	n.a.
**Pixel intensity**				
Foppa *et al*.^35^	Maximum pixel intensity	SPY units	n.r.	n.a.
Protyniak *et al*.^36^	Lowest pixel intensity	0–256 greyscale	66§	n.a.
**Numeric classification score**				
Huh *et al*.^17^	Fluorescence and clinical scoring system	1–5 points	FS 3·5 (range 3–5)	n.a.
Sherwinter *et al*.^19^	Fluorescence and clinical scoring system	1–5 points	n.r.	n.a.

* Values in parentheses are area under the curve, sensitivity (%) and specificity (%).

**Fig. 2:**
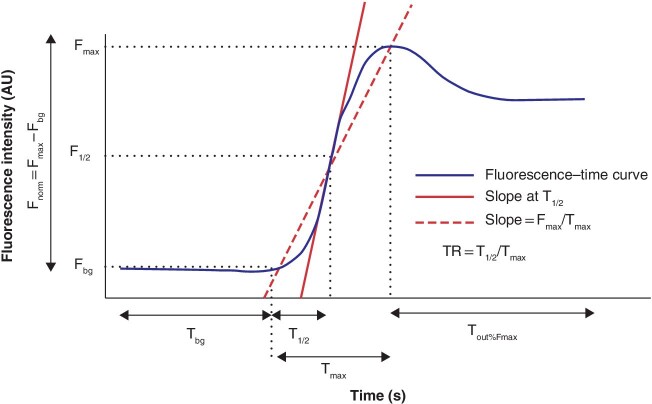
Fluorescence–time curve and its derivatives

AU, arbitrary units; F_norm_, F_max_ corrected for background (F_max_ subtracted by F_bg_); F_max_, maximum intensity; F_bg_, baseline or background intensity; F_1/2_, half of F_max_; T_1/2_, time from ICG inflow to half of F_max_, T_max_, time from ICG inflow to F_max_; TR, time ratio (T_1/2_ divided by T_max_); T_bg_, time from ICG injection to ICG inflow in tissue of interest; T_out_, time of ICG outflow.

† For an explanation of derivatives, see *Fig*. 2.

‡ For patients without anastomotic leakage.

§ Average mean according to volume of procedures. n.a., Not applicable; ICG, indocyanine green; OR, odds ratio; RGEA, right gastroepiploic artery; n.r., not reported; F_max_, maximum intensity; T_max_, time from ICG inflow to F_max_; AU, arbitrary units; T_1/2_, time from ICG inflow to half of F_max_; F_bg_, baseline or background intensity; F_norm_, Fmax corrected for background (F_max_ subtracted by F_bg_); TR, time ratio (T_1/2_ divided by T_max_); T_out_, time of ICG outflow; T_bg_, time from ICG injection to ICG inflow in tissue of interest;; CBR, contrast‐to‐background ratio; FS, fluorescence score.

### Time to fluorescence

#### Manually assessed time to fluorescence

Three studies examined perfusion of the gastric conduit (2 during oesophagectomy in patients and 1 using a porcine oesophagectomy model), one study evaluated perfusion of bowel ends after gastrectomy in patients, and three examined anastomotic perfusion of the lower gastrointestinal tract in patients^14^–^20^. All six human studies^14^^,^^15^^,^^17^–^20^ concerned prospective cohort observations. Change in management and AL were reported in all studies, and management changes were determined by FA in four^14^^,^^15^^,^^18^^,^^20^ of the six studies (*Table* 1). In the porcine oesophagectomy model, ischaemia was studied by reversible ligation of the right gastroepiploic artery.

Five studies^15^–^18^^,^^20^ evaluated time between ICG injection and first enhancement in the bowel ends, one study^19^ observed time between ICG injection and subjective interpreted maximum fluorescent excitation, and one study^14^ evaluated the flow speed (cm/s) of ICG fluorescence through tissue. Mean values for manually assessed time are shown in *Table* 2.

One study^14^ produced a cut‐off value for ICG flow speed (cm/s) to predict AL. Koyanagi and colleagues^14^ calculated the ICG flow speed by evaluating time from first fluorescence in the pylorus to the terminal end of ICG fluorescence divided by the measured distance between the two points. The flow speed was significantly associated with the occurrence of AL, and the cut‐off value was determined as 1·76 cm/s (*Table* 2). In addition, two studies^15^^,^^16^ produced no cut‐off value, but proposed a definition for FA threshold. Kumagai and co‐workers^15^ proposed a ‘90‐second rule’, constructing all anastomoses in the area of the gastric conduit that was enhanced within 90 s (preferably within 60 s) from the first fluorescent enhancement in the root of the right gastroepiploic artery. Only three anastomoses were constructed in an area enhanced after 60 s, of which one anastomosis, constructed in an area enhanced after 77 s, resulted in AL. In another study, Quan *et al*.^16^ defined areas as ischaemic when no fluorescence was seen 360 s after ICG injection.

#### Software‐derived fluorescence–time curves

Seven studies were performed in patients and evaluated perfusion of the gastric conduit during oesophagectomy^21^^,^^22^, perfusion of free jejunal grafts during pharyngo‐oesophagectomy^23^ and perfusion of bowel ends during procedures of the lower gastrointestinal tract.^20^^,^^24^–^26^ The other six studies were animal studies and assessed perfusion in a segment of small bowel or sigmoid in pigs^27^–^31^ or stomach perfusion in pigs^32^.

Six of the seven studies in patients had a prospective design. Four studies^20^^,^^21^^,^^23^^,^^25^ reported change in management according to FA, and the AL rate was observed in five studies^20^–^22^^,^^25^^,^^26^ (*Table* 1). One study^23^ observed venous congestion, which was defined as ‘subjectively’ judged unusually slow fluorescence inflow or graft necrosis due to venous thrombosis, which was confirmed during reoperation. Animal studies observed normal organ perfusion^32^, anastomotic healing^27^ or ischaemic areas after ligation of supplying vessels^28^^–^^31^.

**Fig. 3:**
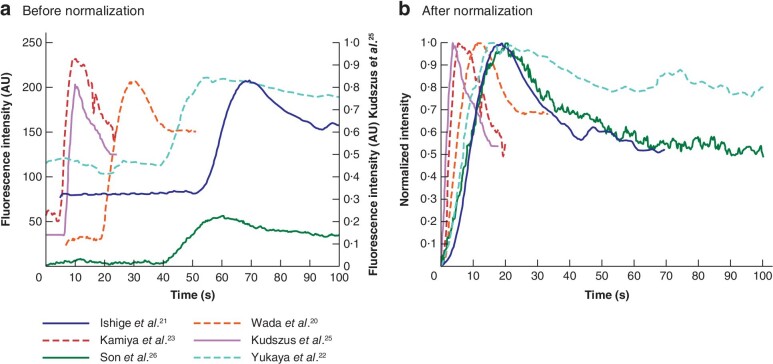
Aggregation of normal fluorescence–time curves before and after normalization of fluorescence intensity and defining *t* = 0

All studies investigated the fluorescence–time curve, which was defined by a software‐derived graph that displayed the fluorescent signal on the *y*‐axis and time on the *x*‐axis of a particular part of the gastrointestinal tract (*Fig*. 2). From this curve, all reported quantitative derivatives are summarized in *Fig*. 2 and their mean values are presented in *Table* 2. A representative ‘normal’ fluorescence–time curve was shown in six of the seven studies in patients^20^^–^^23^^,^^25^^,^^26^. *Fig*. 3*a* shows the raw data of the curves. The baseline intensity (F_bg_) and point *t* = 0 differed for all curves. After intensity normalization of the curves and creating an overlay of *t* = 0, the fluorescence–time curves tended to follow similar morphology, but with a large variation (*Fig*. 3*b*). Of note, when the inflow was steeper, the outflow declined faster. Based on morphology of the fluorescence–time curves, two studies^21^^,^^22^ reported curve types of the gastric conduit during oesophagectomy in patients. Ishige and colleagues^21^ identified ‘normal’ and ‘gradual’ patterns of the curve in six (30 per cent) of 20 and 14 (70 per cent) of 20 cases respectively. However, no AL occurred. Yukaya *et al*.^22^ studied curve types in 27 patients and classified 13 (48 per cent) as normal flow, nine (33 per cent) as delayed inflow, and five (19 per cent) as delayed outflow type. AL occurred in three (23 per cent) of the 13 with normal flow, four (44 per cent) of the nine with delayed inflow, and two (40 per cent) of the five with delayed outflow.

**a** Before and **b** after normalization of fluorescence intensity.

Furthermore, three studies produced cut‐off values for patient outcomes: two^20^^,^^26^ for AL and one^23^ for venous congestion. The cut‐off values were derived for F_max_, T_1/2_, TR and slope (*Table* 2). The studies were inconsistent in considering the quantitative parameters that were predictive for AL. Association of T_1/2_ with AL was evaluated in two studies^20^^,^^26^, and was found to be predictive for AL in one^26^. The slope was predictive for AL in two studies^20^^,^^26^, but with a lower area under the curve in one of the studies^26^.

### Contrast‐to‐background ratio

In this category, one study calculated the CBR during perfusion assessment of the gastric conduit in a porcine oesophagectomy model, and two studies of small bowel segments in pigs and rats^16^^,^^33^^,^^34^. Two different equations for CBR were evaluated. Quan and co‐workers^16^ defined the CBR as fluorescence intensity divided by background fluorescence intensity, and calculated the CBR separately in the gastric conduit and proximal oesophagus. Subsequently, the ratio between the two CBRs was determined (gastric conduit CBR/oesophageal CBR)^16^. Two studies^33^^,^^34^ defined CBR as: (mean fluorescence intensity − mean background fluorescence intensity)/mean background fluorescence intensity. In these two studies, CBR was studied over time. CBR–time curves in small bowel segments appeared to follow a similar shape to that of fluorescence–time curves (*Fig*. 3*b*). Matsui *et al*.^34^ identified four CBR–time curve patterns in pigs: a normal (sharp inflow peak and rapid decline), a delayed (inflow peak and increase over time), a capillary (absent peak and increase over time) and an arterial insufficiency pattern (no change from the background signal). The last two patterns were seen in the ligated areas, whereas the delayed pattern was observed in the adjacent areas. In rats, the absence of an arterial inflow peak in the CBR curve showed accuracy of 85 per cent for predicting clinical necrosis (sensitivity 60 per cent, specificity 100 per cent). In this category, there was scant evidence for a threshold to predict patient outcomes.

### Pixel intensity

Two studies^35^^,^^36^ used embedded software (SPY Elite™ with SPY Q software; Novadaq Technologies, Toronto, Ontario, Canada) to quantify maximum pixel intensity of the ICG fluorescence in patients during bowel resection. Change in management was determined by FA in one study^35^, and AL was observed in the other^36^ (*Table* 1). Quantitative values were reported for the total cohort in one study^36^ (*Table* 2). In this category, there was no evidence for a FA threshold.

### Numeric classification score

In this category, one study^17^ assessed quantification by a numeric classification score in patients undergoing gastric cancer surgery, and one study^19^ by assessing patients undergoing low anterior resection. Change in management, according to conventional white light assessment, and AL were reported in both studies. Sherwinter *et al*.^19^ introduced a scoring system consisting of a fluorescence score of 1–5 (where 1 indicated no uptake and 5 maximum uptake, scoring according to subjective assessment) and a clinical score of 1–5. Huh and colleagues^17^ used the same scoring system; the mean fluorescence score at the stomach side is shown in *Table* 2. In this category, there was scant evidence for a threshold to predict patient outcomes.

## Discussion

In this systematic review all current methods of FA quantification of the ICG fluorescence signal were identified, evaluating perfusion at the anastomotic site during gastrointestinal surgery. The explored quantitative fluorescence parameters were divided into four categories: time to fluorescence, by manually assessed time to fluorescence and by software‐derived fluorescence–time curves; contrast‐to‐background ratio; pixel intensity; and numeric classification score. In the first category, cut‐off values were found for ICG flow speed (cm/s) in the gastric conduit wall and derivatives of the fluorescence–time curves (F_max_, T_1/2_, TR and slope) for AL, and T_1/2_ for venous congestion.

For the short‐term future, manually assessed time to fluorescence seems the most promising method of quantification to produce a threshold for patient outcomes. The method does not require software for analysis, and thus development of a cut‐off value is possible on a large scale. Potentially, time to fluorescence would indicate both arterial and venous problems. Additionally, time to no fluorescence could be of added value to predict tissue necrosis, as suggested by Quan and colleagues^16^. However, this method of quantification is still dependent on the fluorescence intensity and its subjective interpretation. To bypass the fluorescence intensity, fluorescence–time curves seem most promising in the long term. Derivatives of the curve are potentially independent of fluorescence intensity, but also of dose and distance between the imaging system and target organ. The slope, for instance, is distance‐independent. The curves might also specify inadequate arterial inflow or venous outflow^23^^,^^37^. Furthermore, multiple measurements with sequential doses of the fluorescent dye might be possible, even when a high background signal remains after the first FA measurement.

The other categories (contrast‐to‐background ratio, pixel intensity and numeric classification score) seem less appropriate to generate a clinical threshold. Although fluorescence intensity is proportional to the amount of ICG in the tissue, pixel intensity is relative and incomparable, as it depends on patient characteristics, dose, distance, and imaging systems and their settings. The CBR depends on pixel intensity, but also on the background and positioning of the regions of interest. The background differs between imaging systems, as shown in *Fig*. 3, and favourable positioning of regions of interest to calculate CBR can produce bias in the values^38^. The classification score seems easily applicable for clinical purposes, but the assessment of ICG fluorescence is still qualitative.

This systematic review was limited by the low quality of studies and the small number of patient groups with low absolute numbers of AL. Furthermore, data comparison and aggregation was challenging owing to the lack of consensus in definitions in the reporting of FA findings and patient outcomes, standardization of the FA method, calibration of imaging systems, and lack of insight in software algorithms. Below, fundamentals for future research are outlined to overcome these limitations in the future.

Up to now, no consensus exists on the dependent variable of quantification outcomes. Either perfusion characteristics or patient outcomes are now selected as the dependent variable. For example, Nerup and co‐workers^32^ also determined microsphere‐measured regional blood flow, and Diana *et al*.^28^–^31^ measured lactate levels to correlate FA quantification to perfusion characteristics. In this way, quantification of FA will not provide information about the impact on patient outcomes, and a threshold is difficult to produce. In this review, quantitative fluorescence parameters were associated with patient outcomes. However, when considering patient outcomes such as AL, the use of quantitative FA will never account for all the risk factors associated with AL. For future research, it is of paramount importance to investigate quantification of FA in relation to occurrence of AL in a large number of patients, and to correct for other risk factors.

The FA protocol of the studies differed in ICG dose, situation of measurements, field of view, definition of *t* = 0 and change of management. A dose of ICG is recommended at 0·05 mg/kg per bolus and an extra bolus of 2·5 mg if the signal starts to fade, when evaluating literature and experience^5^, ^8^. The field of view would ideally assess the whole organ of interest, but software analysis must be performed in smaller regions to prevent levelling out of the quantitative value. For time to fluorescence, *t* = 0 must be stated clearly. Most studies lacked information on *t* = 0, but, when reported, it was often described as the moment of ICG injection. Using this definition, the location of intravenous access can bias time to fluorescence. It might be more accurate to choose fluorescence enhancement at the base of the supplying vessels of the organ of interest or a nearby organ in the field of view as *t* = 0. Furthermore, change of management in terms of additional resection with adapted level of the anastomotic site due to FA must be reported.

Finally, the available imaging systems have different signal sensitivity due to differences in hardware, optics and image processing, which makes comparison of results difficult. To make data generated by different near‐infrared imaging systems comparable, calibration of the imaging systems could contribute to standardization. Gorpas and colleagues^39^ proposed a composite phantom for calibration of different imaging systems. As well as calibration, validation of the software algorithms is also mandatory to make pooling of data possible. A standard laboratory experiment using the above‐mentioned phantom to assess whether values of the quantitative parameter are the same for different imaging systems and software programs would be helpful, and might allow correction for differences.

In future studies, artificial intelligence might improve the read‐out of FA^40^. Potentially, artificial intelligence will reduce interobserver variation and show new ways to interpret FA. Ideally, artificial intelligence could create a prediction model that combines the clinical history of patients with FA imaging in order to predict AL.

## Funding information

Stryker European Operations

## Supplementary Material

zraa074_Supplementary_Data

## References

[zraa074-B1] van Hagen P , Hulshof MC, van Lanschot JJ, Steyerberg EW, van Berge Henegouwen MI, Wijnhoven BP *et al*.; CROSS Group. Preoperative chemoradiotherapy for esophageal or junctional cancer. N Engl J Med 2012; 366: 2074–2084. 22646630 10.1056/NEJMoa1112088

[zraa074-B2] Borstlap WAA , Westerduin E, Aukema TS, Bemelman WA, Tanis PJ. Anastomotic leakage and chronic presacral sinus formation after low anterior resection: results from a large cross‐sectional study. Ann Surg 2017; 266: 870–877. 28746154 10.1097/SLA.0000000000002429

[zraa074-B3] Grimminger PP , Goense L, Gockel I, Bergeat D, Bertheuil N, Chandramohan SM *et al*. Diagnosis, assessment, and management of surgical complications following esophagectomy. Ann N Y Acad Sci 2018; 1434: 254–273. 29984413 10.1111/nyas.13920

[zraa074-B4] Penna M , Hompes R, Arnold S, Wynn G, Austin R, Warusavitarne J *et al*.; International TaTME Registry Collaborative. Incidence and risk factors for anastomotic failure in 1594 patients treated by transanal total mesorectal excision: results from the International TaTME Registry. Ann Surg 2018; 269: 700–711. 10.1097/SLA.0000000000002653 29315090

[zraa074-B5] van Manen L , Handgraaf HJM, Diana M, Dijkstra J, Ishizawa T, Vahrmeijer AL *et al*. A practical guide for the use of indocyanine green and methylene blue in fluorescence‐guided abdominal surgery. J Surg Oncol 2018; 118: 283–300. 29938401 10.1002/jso.25105 PMC6175214

[zraa074-B6] Schaafsma BE , Mieog JS, Hutteman M, van der Vorst JR, Kuppen PJ, Lowik CW *et al*. The clinical use of indocyanine green as a near‐infrared fluorescent contrast agent for image‐guided oncologic surgery. J Surg Oncol 2011; 104: 323–332. 21495033 10.1002/jso.21943 PMC3144993

[zraa074-B7] Vahrmeijer AL , Hutteman M, van der Vorst JR, van de Velde CJ, Frangioni JV. Image‐guided cancer surgery using near‐infrared fluorescence. Nat Rev Clin Oncol 2013; 10: 507–518. 23881033 10.1038/nrclinonc.2013.123 PMC3755013

[zraa074-B8] Slooter MD , Eshuis WJ, Cuesta MA, Gisbertz SS, van Berge Henegouwen MI. Fluorescent imaging using indocyanine green during esophagectomy to prevent surgical morbidity: a systematic review and meta‐analysis. J Thorac Dis 2019; 11(Suppl 5): S755–S765. 31080655 10.21037/jtd.2019.01.30 PMC6503266

[zraa074-B9] Blanco‐Colino R , Espin‐Basany E. Intraoperative use of ICG fluorescence imaging to reduce the risk of anastomotic leakage in colorectal surgery: a systematic review and meta‐analysis. Tech Coloproctol 2018; 22: 15–23. 29230591 10.1007/s10151-017-1731-8

[zraa074-B10] De Nardi P , Elmore U, Maggi G, Maggiore R, Boni L, Cassinotti E *et al*. Intraoperative angiography with indocyanine green to assess anastomosis perfusion in patients undergoing laparoscopic colorectal resection: results of a multicenter randomized controlled trial. Surg Endosc 2020; 34: 53–60. 30903276 10.1007/s00464-019-06730-0

[zraa074-B11] Krishnan KG , Schackert G, Steinmeier R. The role of near‐infrared angiography in the assessment of post‐operative venous congestion in random pattern, pedicled island and free flaps. Br J Plast Surg 2005; 58: 330–338. 15780227 10.1016/j.bjps.2004.10.003

[zraa074-B12] Moher D , Liberati A, Tetzlaff J, Altman DG; PRISMA Group. Preferred reporting items for systematic reviews and meta‐analyses: the PRISMA statement. BMJ 2009; 339: b2535. 19622551 10.1136/bmj.b2535 PMC2714657

[zraa074-B13] Hooijmans CR , Rovers MM, de Vries RB, Leenaars M, Ritskes‐Hoitinga M, Langendam MW. SYRCLE's risk of bias tool for animal studies. BMC Med Res Methodol 2014; 14: 43. 24667063 10.1186/1471-2288-14-43 PMC4230647

[zraa074-B14] Koyanagi K , Ozawa S, Oguma J, Kazuno A, Yamazaki Y, Ninomiya Y *et al*. Blood flow speed of the gastric conduit assessed by indocyanine green fluorescence: new predictive evaluation of anastomotic leakage after esophagectomy. Medicine 2016; 95: e4386. 10.1097/MD.0000000000004386 PMC5265869 27472732

[zraa074-B15] Kumagai Y , Hatano S, Sobajima J, Ishiguro T, Fukuchi M, Ishibashi K‐I *et al*. Indocyanine green fluorescence angiography of the reconstructed gastric tube during esophagectomy: efficacy of the 90‐second rule. Dis Esophagus 2018; 31: doy052. 10.1093/dote/doy052 29897432

[zraa074-B16] Quan YH , Kim M, Kim HK, Kim BM. Fluorescent image‐based evaluation of gastric conduit perfusion in a preclinical ischemia model. J Thorac Dis 2018; 10: 5359–5367. 30416783 10.21037/jtd.2018.08.46 PMC6196182

[zraa074-B17] Huh YJ , Lee HJ, Kim TH, Choi YS, Park JH, Son YG *et al*. Efficacy of assessing intraoperative bowel perfusion with near‐infrared camera in laparoscopic gastric cancer surgery. J Laparoendosc Adv Surg Tech Part A 2019; 29: 476–483. 10.1089/lap.2018.0263 30589374

[zraa074-B18] Kim JC , Lee JL, Park SH. Interpretative guidelines and possible indications for indocyanine green fluorescence imaging in robot‐assisted sphincter‐saving operations. Dis Colon Rectum 2017; 60: 376–384. 28267004 10.1097/DCR.0000000000000782

[zraa074-B19] Sherwinter DA , Gallagher J, Donkar T. Intra‐operative transanal near infrared imaging of colorectal anastomotic perfusion: a feasibility study. Colorectal Dis 2013; 15: 91–96. 22632448 10.1111/j.1463-1318.2012.03101.x

[zraa074-B20] Wada T , Kawada K, Takahashi R, Yoshitomi M, Hida K, Hasegawa S *et al*. ICG fluorescence imaging for quantitative evaluation of colonic perfusion in laparoscopic colorectal surgery. Surg Endosc 2017; 31: 4184–4193. 28281123 10.1007/s00464-017-5475-3

[zraa074-B21] Ishige F , Nabeya Y, Hoshino I, Takayama W, Chiba S, Arimitsu H *et al*. Quantitative assessment of the blood perfusion of the gastric conduit by indocyanine green imaging. J Surg Res 2019; 234: 303–310. 30527489 10.1016/j.jss.2018.08.056

[zraa074-B22] Yukaya T , Saeki H, Kasagi Y, Nakashima Y, Ando K, Imamura Y *et al*. Indocyanine green fluorescence angiography for quantitative evaluation of gastric tube perfusion in patients undergoing esophagectomy. J Am Coll Surg 2015; 221: e37–e42. 26206660 10.1016/j.jamcollsurg.2015.04.022

[zraa074-B23] Kamiya K , Unno N, Miyazaki S, Sano M, Kikuchi H, Hiramatsu Y *et al*. Quantitative assessment of the free jejunal graft perfusion. J Surg Res 2015; 194: 394–399. 25472574 10.1016/j.jss.2014.10.049

[zraa074-B24] Bornstein JE , Munger JA, Deliz JR, Mui A, Chen CS, Kim S *et al*. Assessment of bowel end perfusion after mesenteric division: eye *versus* SPY. J Surg Res 2018; 232: 179–185. 30463716 10.1016/j.jss.2018.06.015

[zraa074-B25] Kudszus S , Roesel C, Schachtrupp A, Hoer JJ. Intraoperative laser fluorescence angiography in colorectal surgery: a noninvasive analysis to reduce the rate of anastomotic leakage. Langenbecks Arch Surg 2010; 395: 1025–1030. 20700603 10.1007/s00423-010-0699-x

[zraa074-B26] Son GM , Kwon MS, Kim Y, Kim J, Kim SH, Lee JW. Quantitative analysis of colon perfusion pattern using indocyanine green (ICG) angiography in laparoscopic colorectal surgery. Surg Endosc 2019; 33: 1640–1649. 30203201 10.1007/s00464-018-6439-y PMC6484815

[zraa074-B27] Nerup N , Ring LL, Strandby RB, Egeland C, Svendsen MBS, Hasselby JP *et al*. Quantitative perfusion assessment of intestinal anastomoses in pigs treated with glucagon‐like peptide 2. Langenbecks Arch Surg 2018; 403: 881–889. 30338374 10.1007/s00423-018-1718-6

[zraa074-B28] Diana M , Agnus V, Halvax P, Liu YY, Dallemagne B, Schlagowski AI *et al*. Intraoperative fluorescence‐based enhanced reality laparoscopic real‐time imaging to assess bowel perfusion at the anastomotic site in an experimental model. Br J Surg 2015; 102: e169–e176. 25627131 10.1002/bjs.9725

[zraa074-B29] Diana M , Dallemagne B, Chung H, Nagao Y, Halvax P, Agnus V *et al*. Probe‐based confocal laser endomicroscopy and fluorescence‐based enhanced reality for real‐time assessment of intestinal microcirculation in a porcine model of sigmoid ischemia. Surg Endosc 2014; 28: 3224–3233. 24935199 10.1007/s00464-014-3595-6

[zraa074-B30] Diana M , Halvax P, Dallemagne B, Nagao Y, Diemunsch P, Charles AL *et al*. Real‐time navigation by fluorescence‐based enhanced reality for precise estimation of future anastomotic site in digestive surgery. Surg Endosc 2014; 28: 3108–3118. 24912446 10.1007/s00464-014-3592-9

[zraa074-B31] Diana M , Noll E, Diemunsch P, Dallemagne B, Benahmed MA, Agnus V *et al*. Enhanced‐reality video fluorescence: a real‐time assessment of intestinal viability. Ann Surg 2014; 259: 700–707. 23532109 10.1097/SLA.0b013e31828d4ab3

[zraa074-B32] Nerup N , Andersen HS, Ambrus R, Strandby RB, Svendsen MBS, Madsen MH *et al*. Quantification of fluorescence angiography in a porcine model. Langenbecks Arch Surg 2017; 402: 655–662. 27848028 10.1007/s00423-016-1531-z

[zraa074-B33] Ashitate Y , Vooght CS, Hutteman M, Oketokoun R, Choi HS, Frangioni JV. Simultaneous assessment of luminal integrity and vascular perfusion of the gastrointestinal tract using dual‐channel near‐infrared fluorescence. Mol Imaging 2012; 11: 301–308. 22954146 PMC3439161

[zraa074-B34] Matsui A , Winer JH, Laurence RG, Frangioni JV. Predicting the survival of experimental ischaemic small bowel using intraoperative near‐infrared fluorescence angiography. Br J Surg 2011; 98: 1725–1734. 21953541 10.1002/bjs.7698 PMC3235697

[zraa074-B35] Foppa C , Denoya PI, Tarta C, Bergamaschi R. Indocyanine green fluorescent dye during bowel surgery: are the blood supply ‘guessing days’ over? Tech Coloproctol 2014; 18: 753–758. 24558047 10.1007/s10151-014-1130-3

[zraa074-B36] Protyniak B , Dinallo AM, Boyan WP Jr, Dressner RM, Arvanitis ML. Intraoperative indocyanine green fluorescence angiography – an objective evaluation of anastomotic perfusion in colorectal surgery. Am Surg 2015; 81: 580–584. 26031270 10.1177/000313481508100621

[zraa074-B37] Quero G , Lapergola A, Barberio M, Seeliger B, Saccomandi P, Guerriero L *et al*. Discrimination between arterial and venous bowel ischemia by computer‐assisted analysis of the fluorescent signal. Surg Endosc 2019; 33: 1988–1997. 30327913 10.1007/s00464-018-6512-6

[zraa074-B38] Hoogstins C , Burggraaf JJ, Koller M, Handgraaf H, Boogerd L, van Dam G *et al*. Setting standards for reporting and quantification in fluorescence‐guided surgery. Mol Imaging Biol 2019; 21: 11–18. 29845427 10.1007/s11307-018-1220-0

[zraa074-B39] Gorpas D , Koch M, Anastasopoulou M, Bozhko D, Klemm U, Nieberler M *et al*. Multi‐parametric standardization of fluorescence imaging systems based on a composite phantom. IEEE Trans Biomed Eng 2020; 67: 185–192. 30990172 10.1109/TBME.2019.2910733

[zraa074-B40] Topol EJ . High‐performance medicine: the convergence of human and artificial intelligence. Nat Med 2019; 25: 44–56. 30617339 10.1038/s41591-018-0300-7

