# Supplementary material for: Defining indocyanine green fluorescence to assess anastomotic perfusion during gastrointestinal surgery: systematic review
Source: BJS Open. 2021 Apr 24;5(2):zraa074. doi: 10.1093/bjsopen/zraa074 (PMC8271268; doi:10.1093/bjsopen/zraa074)
Supplement: zraa074_Supplementary_Data [file zraa074_supplementary_data.docx]

BJS5_50296

**Defining indocyanine green fluorescence to assess anastomotic perfusion during gastrointestinal surgery: systematic review**

**M. D. Slooter, M. S. E. Mansvelders, P. R. Bloemen, S. S. Gisbertz, W. A. Bemelman, P. J. Tanis, R. Hompes, M. I. van Berge Henegouwen and D. M. de Bruin**

**Appendix S1** PubMed search term

perfusion[tiab] AND ("Indocyanine Green"[Mesh] OR "Fluorescence"[Mesh] OR fluorescence[tiab] OR indocyanine green[tiab] OR fluorescence angiograph*[tiab] OR fluorescence imaging[tiab]) AND
(quantification[tiab] OR quantitative[tiab] OR assessment[tiab] OR confirm*[tiab])

**Table S1** Title and abstract screening criteria

| **Inclusion** | **Exclusion** |
| --- | --- |
| Perfusion assessment using ICG^a^ | Perfusion assessment with other dye than ICG^a^ |
| Analysis of fluorescence is done quantitatively^b^ | ICG^a^ for hepatic clearance |
| Gastrointestinal surgery | Qualitatively, but not quantitatively analysed |
| All research levels^c^ | Language other than English |
| ^a^ Indocyanine Green | |
| ^b^ 'Quantitative' mentioned in the title or abstract, or description of quantitative parameter | |
| ^c^ Laboratory experiments, animal and human studies | |

**Table S2** Quality assessment of human studies

| Reference | | Selection | | | | | Comparability | | Outcome | | | | Total Score |
| --- | --- | --- | --- | --- | --- | --- | --- | --- | --- | --- | --- | --- | --- |
|  |  | 1) | 2) | 3) | 4) | **Score** | 1) | **Score** | 1) | 2) | 3) | **Score** |  |
| Upper GI | Huh et al. 2018 | a | c | a | a | 3/4 | - | 0/2 | b | a | a | 3/3 | Poor |
|  | Ishige et al. 2019 | a | c | a | a | 3/4 | - | 0/2 | b | a | a | 3/3 | Poor |
|  | Kamiya et al. 2014 | a | c | a | a | 3/4 | - | 0/2 | b | a | a | 3/3 | Poor |
|  | Koyanagi et al. 2016 | a | c | a | a | 3/4 | - | 0/2 | b | a | a | 3/3 | Poor |
|  | Kumagai et al. 2018 | a | c | a | a | 3/4 | - | 0/2 | b | a | a | 3/3 | Poor |
|  | Yukaya et al. 2015 | a | c | a | a | 3/4 | - | 0/2 | b | a | a | 3/3 | Poor |
|  |  |  |  |  |  |  |  |  |  |  |  |  |  |
| Lower GI | Bornstein et al. 2018 | a | c | a | a | 3/4 | - | 0/2 | b | a | a | 3/3 | Poor |
|  | Foppa et al. 2014 | a | c | c | a | 2/4 | - | 0/2 | c | a | c | 2/3 | Poor |
|  | Kim et al. 2017 | a | a | a | a | 4/4 | a,b | 2/2 | b | a | a | 3/3 | Good |
|  | Kudszus et al. 2010 | a | a | a | a | 4/4 | a,b | 2/2 | b | a | a | 3/3 | Good |
|  | Protyniak et al. 2015 | a | c | a | a | 3/4 | - | 0/2 | b | a | a | 3/3 | Poor |
|  | Sherwinter et al. 2012 | a | c | a | a | 3/4 | - | 0/2 | b | a | a | 3/3 | Poor |
|  | Son et al. 2018 | a | c | a | a | 3/4 | - | 0/2 | b | a | a | 3/3 | Poor |
|  | Wada et al. 2017 | a | c | a | a | 3/4 | - | 0/2 | b | a | a | 3/3 | Poor |

**Table S3** Quality assessment of animal studies

| **Type of bias** | | **Selection** | | | **Performance** | | **Detection** | | **Attrition** | **Reporting** | **Other** |
| --- | --- | --- | --- | --- | --- | --- | --- | --- | --- | --- | --- |
| **Reference** | | Sequence generation | Baseline characteristics | Allocation concealment | Random housing | Blinding | Random outcome assessment | Blinding | Incomplete outcome data | Selective outcome reporting | Other sources of bias |
| **Upper GI** | Nerup et al. 2016 | x | x | x | ? | x | ? | x | ✓ | ✓ | ✓ |
|  | Quan et al. 2018 | x | x | x | ? | x | ? | x | ✓ | ✓ | ✓ |
|  |  |  |  |  |  |  |  |  |  |  |  |
| **Lower GI** | Ashitate et al. 2012 | x | x | x | ? | x | ? | x | ✓ | ✓ | ✓ |
|  | Diana et al. June 10 2014 | ? | x | x | ? | x | ? | ✓ | ✓ | ✓ | ✓ |
|  | Diana et al. June 17 2014 | x | x | x | ? | x | ? | ✓ | ✓ | ✓ | ✓ |
|  | Diana et al. April 2014 | x | x | x | ? | x | ? | x | ✓ | ✓ | ✓ |
|  | Diana et al. 2015 | x | x | x | ? | x | ? | x | ✓ | ✓ | ✓ |
|  | Matsui et al. 2011 | x | x | x | ? | x | ? | ? | ✓ | ✓ | ✓ |
|  | Nerup et al. 2018 | ✓ | ✓ | ✓ | ? | ✓ | ? | ✓ | ✓ | ✓ | ✓ |

**Table S4** Characteristics of human studies

|  | **Reference** | **Study design** | **ICGᵅ group (n= (Male : Female))** | **Age (years, mean 土SD)** | **Surgical procedure** | **ICGᵅ measurement** | **ICGᵅ dose (mg/bolus)** | **Camera system** | **Software program** |
| --- | --- | --- | --- | --- | --- | --- | --- | --- | --- |
|  |  |  |  |  |  | **Before or after anastomosis / Serosa or mucosa** |  |  |  |
| **Upper GI** | Huh et al. 2018 | Prospective | 30 (18:12) | 58.7 土11.0 | Gastrectomy * | After anastomosis / serosa | 2.5-5 | Karl Storz Endoskope spiesᴬ | N.A.ᵉ |
|  | Ishige et al. 2019 | Prospective | 20 (19 : 1) | Median: 71 (range 65-85) | Esophagectomy with gastric conduit reconstruction | Before vessel disconnection, before, after anastomosis / Serosa | 1.25 | Olympusᴮ connected to endocopy^C^ | ROIsᴰ |
|  | Kamiya et al. 2014 | Prospective | 26 (24 : 2) | 63.1 (49-75) | Pharyngoesophagectomy with FJGᵇ transfer | Before anastomosis / serosa | 5 | Photodynamic Eyeᴰ | ROIsᴰ |
|  | Koyanagi et al. 2016 | Prospective | 40 (34 : 6) | Median: 68 (26-82) | Esophagectomy with gastric conduit reconstruction | Before anastomosis / serosa | 1.25, 2.5 | Photodynamic Eyeᴰ | N.A.ᵉ |
|  | Kumagai et al. 2018 | Prospective | 70 (59 : 11) | Median: 71 (46-82) | Esophagectomy with gastric conduit reconstruction | Before anastomosis / serosa | 2.5 | Photodynamic Eyeᴰ | N.A.ᵉ |
|  | Yukaya et al. 2015 | Prospective | 27 (26:1) | Median: 66.8 (range 40-86) | Esophagectomy with gastric conduit reconstruction | Before anastomosis / serosa | 0.1 mg/kg | Hyper Eye Medical Systemᴱ | LumiViewᴷ |
|  | | | | | | | | | |
| **Lower GI** | Bornstein et al. 2018 | Prospective | 46 (23 : 23) | 48.2 土16.8 | Bowel resections** | Before anastomosis / serosa | N.R.ᵈ | SPY Elite™^F^ | CAPA platform |
|  | Foppa et al. 2014 | Prospective | 4/160 (3 : 1) | 67.5 土11.8 | Bowel resections *** | Before anastomosis / serosa | 0.1-0.3 mg/kg | SPY Elite™ᴳ | SPY-Q F |
|  | Kim et al. 2017 | Retrospective | 310 (182 : 128) | 58 土11 | (Ultra-)LARᶜ | Before and after anastomosis / serosa | 10 | Da Vinci Si or Xiᴴ | N.A.ᵉ |
|  | Kudszus et al. 2010 | Retrospective | 201 (85 : 116) | 67.8 土25.2 | Colorectal cancer resections **** | Before anastomosis / Serosa | 0.2-0.5 mg/kg | IC-View®ᴵ | IC-Calc® |
|  | Protyniak et al. 2015 | Retrospective | 77 (34 : 43) | 61.8 | Colorectal resections ***** | After anastomosis / serosa | N.R.ᵈ | SPY Elite™^F^ | SPY-Q^F^ |
|  | Sherwinter et al. 2012 | Prospective | 20 (6 : 14) | 67.2 土14.9 | LARᶜ | After anastomosis / mucosa | 2.5 | Pinpoint^F^ with Custom built introducerᴶ | N.A.ᵉ |
|  | Son et al. 2018 | Prospective | 86 (63 : 23) | 65.4 | (L)ARᶜ | Before anastomosis / serosa | 0.25 mg/kg | IMAGE1 S™ᴬ | Tracker 4.97ᴸ |
|  | Wada et al. 2017 | Retrospective | 112 (81 : 31) | Median: 67 (28-89) | Left sided colorectal cancer resection ****** | Before anastomosis / serosa | 5 | Photodynamic Eyeᴰ | ROIsᴰ |

ᵅIndocyanine Green, ᵇFree jejunal graft, ᶜ(Low) anterior resection, ᵈnot reported, ᵉnot applicable

*Gastroduodenostomy, gastrojejunostomy, total gastrectomy, pylorus-preserving gastrectomy. **Small bowel resection, ileostomy reversal, ileostomy formation, ileostomy/kock-pouch revision, ileocolic resection/right hemicolectomy, anterior/segmental/subtotal colon resection, low anterior resection, colostomy revision. ***Small bowel resection, proctocolectomy, low anterior resection, Hartmann reversal. ****Right hemicolon, segmental colonic resection, left hemicolon and rectum. *****Ileocolic, transverse, right, left sigmoid, low anterior resection. ******Sigmoidectomy, high anterior resection, low anterior resection.

ᴬKarl Storz TM GmbH & Co. KG, Tuttlingen, Germany. ᴮOlympys, Tokyo, Japan. ^C^DH28R, Fujifilm, Japan. ᴰHamamatsu Photonics K.K., Hamamatsu, Japan, ᴱMizuho Corporation. ^F^Novadaq Technologies. Toronto, Canada. ᴳLifecell Corporation, NJ, USA. ᴴFirefly, Intuitive Surgical, Sunnyvale, CA, USA. ᴵPulsion Medical Systems AG, Munich, Germany. ᴶApplied Medical, Rancho Santa Margarita, CA, USA. ᴷMizuho Corporation.

**Table S5** Characteristics of animal studies

|  | **Reference** | **ICGᵅ group (n=)** | **Surgical procedure** | **ICGᵅ dose (mg/kg/bolus)*** | **Camera system** | **Software program** |
| --- | --- | --- | --- | --- | --- | --- |
| **Upper GI** | Nerup et al. 2016 | 7 pigs | Midline laparotomy (stomach perfusion) | 0.39 | IMAGE1™ᴬ | Custom made software |
|  | Quan et al. 2018 | 10 pigs | Esophagectomy with gastric conduit reconstruction | 0.6 | Custom-manufactured ICFIS | Steampix6ᴮ |
|  |  | | | | | |
| **Lower GI** | Ashitate et al. 2012 | 4 pigs | Jejunojenunostomy, creation of ischemic small bowel segment | 10 ml of 80uM /bolus | FLARE™ | Custom made software |
|  | Diana et al. April 2014 | 7 pigs | Creation of ischemic small bowel segments | 0.5 | D-Light P laparoscopeᴬ | VR-RENDER PERFUSION |
|  | Diana et al. June 10 2014 | 6 pigs | Creation of ischemic small bowel segments | 0.5 | D-Light P laparoscopeᴬ | ER-PERFUSION^C^ |
|  | Diana et al. June 17 2014 | 6 pigs | Creation of ischemic sigmoid segment | 0.5 | D-Light P laparoscopeᴬ | ER-PERFUSION^C^ |
|  | Diana et al. 2015 | 13 pigs | Creation of ischemic small bowel segments | 0.5 | D-Light P laparoscopeᴬ | ER-PERFUSION^C^ |
|  | Matsui et al. 2011 | 24 pigs, 60 rats | Creation of ischemic small bowel segment | Pigs 0.05,  rats 0.15 | FLARE™ | N.A.ᵇ |
|  | Nerup et al. 2018 | 10 pigs | Small bowel anastomoses | 0.25 | IMAGE1™ᴬ | Custom made software |

*all serosal measurements

ᵅIndocyanine Green, ᵇnot applicable

ᴬKarl Storz GmbH & Co. KG, Tuttlingen, Germany. ᴮNorpix, Canada. ^C^IRCAD, Strasbourg, France.
